# Positive Impulse Phase versus Propulsive Impulse Phase: Correlations between Asymmetry and Countermovement Jump Performance

**DOI:** 10.3390/jfmk7020031

**Published:** 2022-04-05

**Authors:** Keith B. Painter, William Guy Hornsby, Kevin Carroll, Satoshi Mizuguchi, Michael H. Stone

**Affiliations:** 1 Department of Sport Physiology & Performance, East Tennessee State University, Johnson City, TN 37614, USA; carrollk@etsu.edu (K.C.); mizuguchi@etsu.edu (S.M.); stonem@etsu.edu (M.H.S.); 2 Department of Coaching and Teaching Studies, College of Physical Activity and Sport Sciences, West Virginia University, Morgantown, WV 26505, USA; william.hornsby@mail.wvu.edu

**Keywords:** asymmetry, symmetry, ground reaction force, jumping performance, positive impulse

## Abstract

The relationship between asymmetry and performance is still undetermined in the literature. Methods of assessing asymmetry have been inconsistent and focused on the analysis of jumping asymmetry. Dual ground reaction forces are prevalent in athlete monitoring, though underutilized in asymmetry research. The purpose of this study was to assess the relationship of countermovement jump (CMJ) impulse asymmetry to performance in collegiate soccer athletes. Male and female athletes were selected from an ongoing athlete research repository database of NCAA D-I soccer athletes. All athletes contributed two maximal effort unweighted (CMJ0) and weighted countermovement jumps (CMJ20) using the mean for calculations. Propulsive phase asymmetry scores (PrPAS) and positive impulse asymmetry scores (PIAS) were calculated to determine the magnitude of asymmetry for each prospective phase. Statistically significant correlations were found between CMJ0 jump height and unweighted PIAS (*r* = −0.43) in females. Males had statistically significant correlations between CMJ20 jump height and weighted PIAS (*r* = −0.49). Neither unweighted PrPAS nor weighted PrPAS produced statistically significant correlations (*r* < 0.26) to their prospective jump heights. When assessing CMJ asymmetry, it is recommended to conduct both weighted and unweighted CMJ testing, utilizing PIAS as the metric to be assessed.

## 1. Introduction

Asymmetry can be defined as the unequal split of two halves whether that be side-to-side, front-to-back or some other combination. Some level of asymmetry is expected in all human movements and there is likely a threshold for meaningful asymmetry [1]. As such, sporting asymmetry refers to developed asymmetries to match the demands of a particular sport [2,3,4], indicating that athletes likely learn to adapt to asymmetrical developments and use it to their advantage. This may account for the task dependent nature of asymmetry [3,5,6], which should be considered when investigating asymmetry with performance, as motor coordination may be more indicative of poor performance than strength asymmetry [7].

**Figure 1:**
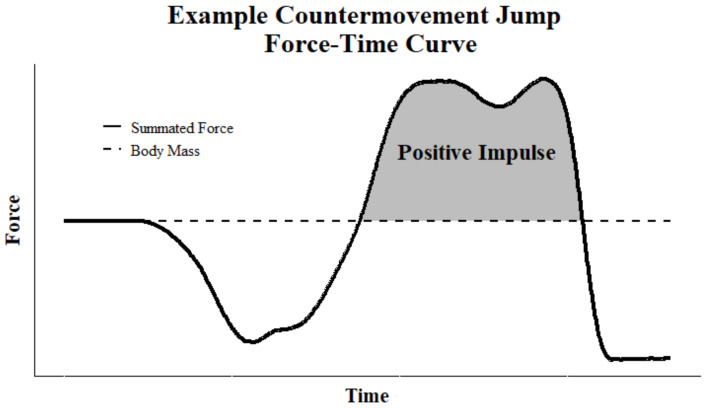
Positive Impulse Example Illustration.

Asymmetry and performance have shown mixed results in the literature and there has not been a consensus on their relationship [3]. Some studies have provided evidence that various jumping asymmetries do relate to performance measures [2,8,9,10], others refute those claims [11,12]. Most studies of ground reaction force asymmetry have focused on the peaks associated with the propulsive phase of the countermovement jump (CMJ) [13,14], which leaves out the unweighting and breaking phases that account for over half of the jump. These phases are likely more malleable when encountering asymmetry [5]. While the unweighting phase has been shown to be less reliable with self-selected depths [15], the breaking phase has been shown to produce good to excellent reliability [16]. The breaking phase asymmetry could also be an indicator of how an athlete recovers and reacts to the unweighting phase. For these reasons, this correlational study will deal with an assessment of the propulsive phase and the positive impulse phase, which is comprised of the breaking phase to the point of return to system mass just before take-off (see Figure 1), which is also the impulse due to the dual ground reaction force (dGRF) [17].

Additionally, very few researchers have included weighted jumps during asymmetry investigations, and this may be a crucial but overlooked component. Bilateral weighted CMJs may illicit a similar asymmetry response compared to previously investigated unilateral drop jumps [9] and they have been found to exacerbate unloaded jump asymmetry values [5]. Assessing loaded CMJs may be more representative of loads experienced in practice and game situations (i.e., high speed change of direction), as well as a method of increasing the difficultly of a learned task [18]. Adding a load should either cause athletes to become more or less asymmetrical in the time it takes to achieve PF. Thus, the inclusion of weighted CMJ asymmetry was also assessed. Overall, the goal of this study is to assess the relationship of CMJ asymmetry to CMJ performance using two alternative methods: propulsive phase asymmetry only and positive impulse phase asymmetry. We hypothesize that the positive phase asymmetry will have a greater relationship to performance than the propulsive phase asymmetry alone.

## 2. Materials and Methods

This was a retrospective study examining the relationships of jumping asymmetry with performance. All data were selected from the ongoing athlete monitoring research repository database of NCAA D-I male and female soccer teams. The inclusion criteria for each athlete were as follows: (1) all jump testing was conducted on dual force plates; (2) participated in all performance tests during the testing session with a minimum of two maximum effort trials for each jump test; (3) tests were conducted during the pre-season phase during the same month. A total of 59 out of 70 soccer athletes were selected (male *n* = 35, female *n* = 24) after implementing the inclusion criteria. This research was approved by the East Tennessee State University Institutional Review Board (IRB reference number: c0719.17sw).

### 2.1. Testing Protocols

All athletes were cleared to participate by the athletic training staff and participated in the same athlete monitoring protocols. Testing consisted of a urinary specific gravity hydration test, anthropometrics, standard warm up, unweighted countermovement jumps (CMJ0), weighted countermovement jumps (CMJ40), and isometric mid-thigh pulls (IMTP). Athletes were given a 50% and a 75% of perceived maximum effort warm up before each test and given a 30 s rest time between jumps. Athletes were given up to seven trials for each jump type (CMJ0, CMJ40) with the trials ending when the athlete produced two jump heights within 2.0 cm of each other. The average of the two trials was used for data analysis to reduce the potential for measurement error. All CMJ40 attempts were performed with a 20 kg barbell (behind neck, across shoulders) and CMJ0 were performed with a PVC pipe in place of the barbell to maintain the position and prevent arm swing. Before the start of each jump type, a standing system mass value was obtained for a minimum of 1.0 s. All jumps had an athlete self-selected unweighting depth. All IMTP assessments were conducted after jump testing was complete to assess overall strength. Athletes were positioned in a customized stationary rack at 125 ± 5° knee angle with an upright trunk position [18,19,20]. All jumps and IMTPs were performed on dual force plates (91.0 cm × 45.5 cm; Rice Lake Weighing Systems, Rice Lake, WI, USA) with the analog signal from the force platform collected using a customized LabView (National Instruments, Austin, TX, USA) program at 1000 Hz.

### 2.2. Data Analysis

The vertical dGRF data from jumps were exported as text files and analyzed using a customized 2019 Microsoft Excel^®^ spreadsheet with VBA coding (Microsoft Corporations, Redmond, WA, USA) adopting previously established methods of analyzing CMJs [16,21]. Raw voltage data from force plates were smoothed using a 50-point finite impulse response filter and then converted into Newtons (N) to develop the force-time curve (F-Tc) for each jump.

The propulsive phase of the CMJ is defined as the end of the eccentric phase to the start of flight time [22]. Using impulse, a propulsive phase asymmetry score (PrPAS) and a positive impulse asymmetry score (PIAS) were calculated for each jump using an absolute asymmetry equation (see Symmetry Index Equation) [22,23] to assess the overall magnitude of asymmetry using a percentage. This allowed for a true assessment of asymmetry rather than focusing on which side was dominant since previous research has shown asymmetry may shift sides depending on the task [3]. 

Symmetry Index Equation:

(1) $$\frac{\left(Maximum\;value-Minimum\;value\right)}{\left(Maximum\;value+Minimum\;value\right)}\times 100.$$

### 2.3. Statistics

Means and standard deviations are presented as *M* ± *SD*. Normality of distribution was checked using the Shapiro–Wilk tests. Welch’s *t*-tests were calculated to assess male to female differences. A Pearson’s product correlation (*r*) with 95% confidence intervals (CI) was computed between all tests and asymmetry and is reported as *r* [CI]. Critical *r* values were calculated for both males and females to determine the minimal level of significant *r* values. Additionally, *R*^2^ was reported to illuminate the proportion of explained variance for each metric of interest. An alpha of 0.05 was set for all applicable statistical analyses. Data were analyzed using a customized 2019 Microsoft Excel^®^ spreadsheet (Microsoft Corporations, Redmond, Washington, WA, USA).

## 3. Results

Descriptive statistics can be found in Table 1. All performance variables were normally distributed; however, asymmetry scores were not. Due to the overall curvilinear nature of the scatter plots from the concentration of asymmetry values at the lower end of the spectrum, a natural log transformation was used. After applying a natural log transformation to asymmetry scores, they were found to be normally distributed. Additionally, no outliers or influential cases were identified.

Statistically significant correlations were found between unweighted positive impulse asymmetry score (PIAS0) and weighted positive impulse asymmetry score (PIAS20) for males and females (*r* = 0.84, 0.87; respectively). While unweighted propulsive phase asymmetry score (PrPAS0) to weighted propulsive phase asymmetry score (PrPAS20) were also statistically significant, the correlation was not as great for males (*r* = 0.51) nor for females (*r* = 0.57).

Males and females produced statistically significant differences between unweighted jump heights (JH0) (*p* < 0.01), weighted jump heights (JH20) (*p* < 0.01), body mass (BM) (*p* = 0.01), and allometrically scaled isometric peak force (IPFa) (*p* < 0.01). No statistically significant differences were found between the sexes for PrPAS0 (*p* = 0.97), PrRAS20 (*p* = 0.67), PIAS0 (*p* = 0.44), nor PIAS20 (*p* = 0.20). Critical *r* values were determined to be ± 0.33 for males and ± 0.40 for females.

**Figure 2:**
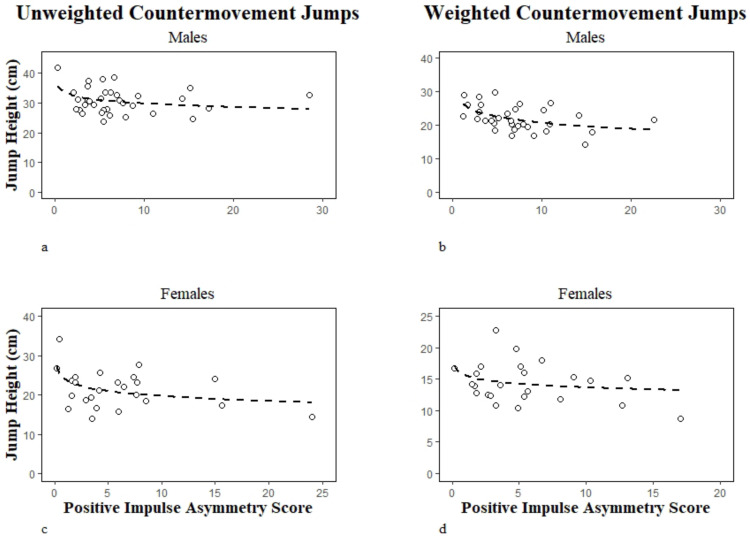
Plotted Countermovement Jumps with Positive Impulse Asymmetry Score; (**a**) Male Unweighted Countermovement Jumps; (**b**) Male Weighted Countermovement Jumps; (**c**) Female Unweighted Countermovement Jumps; (**d**) Female Weighted Countermovement Jumps.

Unweighted jump heights for males (see Figure 2) fell just short of statistical significance with PIAS0. However, females (see Figure 2) did have statistically significant correlations with PIAS0 (see Table 2). Conversely, males presented statistically significant correlations between weighted jump heights and PIAS20 (see Table 2 and Figure 2), but females did not reach statistical significance in the same (Figure 2). 

No statistically significant correlations were found for males or females between PrPAS0 and PrPAS20 with their respective jump heights (see Table 3). Interestingly, three of the four correlations became positive. 

**Figure 3:**
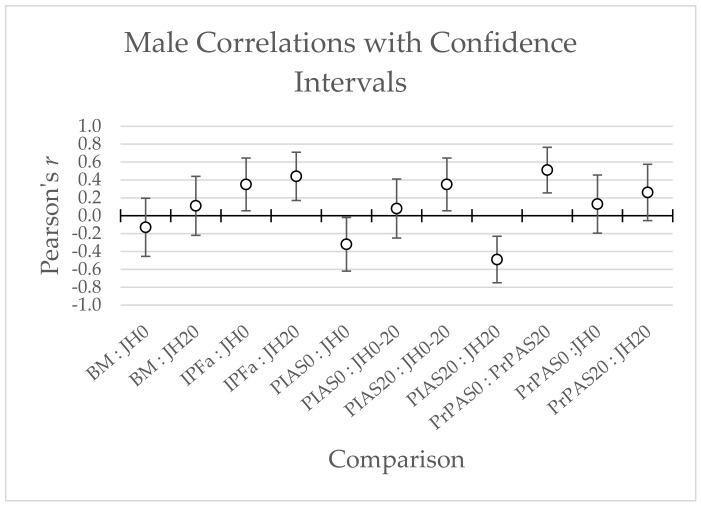
Male Correlations with Confidence Intervals; BM = Body Mass; JH0 = Unweighted Jump Height; JH20 = Weighted Jump Height; JH0-20 = Percent drop-off from unweighted jump height to weighted jump height; IPFa = Allometrically Scaled Isometric Mid-thigh Pull Peak Force; PIAS0 = Unweighted positive impulse asymmetry score; PIAS20 = Weighted positive impulse asymmetry score; PrPAS0 = Unweighted propulsive phase impulse asymmetry score; PrPAS20 = Weighted propulsive phase impulse asymmetry score.

**Figure 4:**
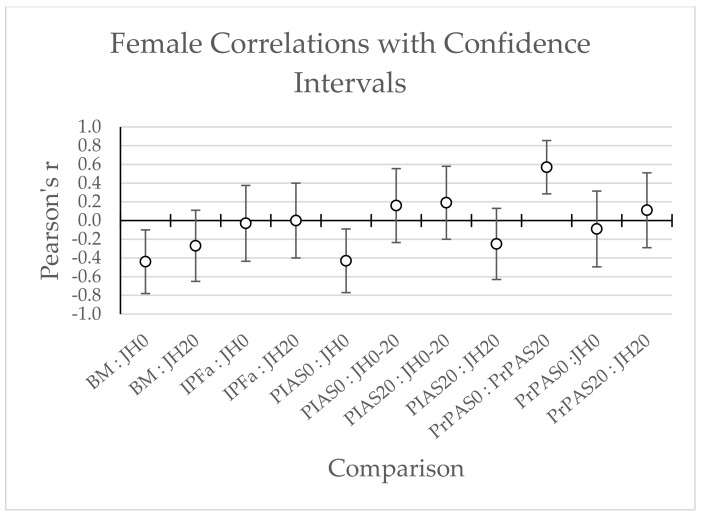
Female Correlations with Confidence Intervals; BM = Body Mass; JH0 = Unweighted Jump Height; JH20 = Weighted Jump Height; JH0-20 = Percent drop-off from unweighted jump height to weighted jump height; IPFa = Allometrically Scaled Isometric Mid-thigh Pull Peak Force; PIAS0 = Unweighted positive impulse asymmetry score; PIAS20 = Weighted positive impulse asymmetry score; PrPAS0 = Unweighted propulsive phase impulse asymmetry score; PrPAS20 = Weighted propulsive phase impulse asymmetry score.

No statistically significant correlations were found between PrPSA0, PrPSA20, PIAS0, nor PIAS20 with IPFa in males (*r* = 0.15 [0.09, 0.21], 0.22 [0.17, 0.28], 0.00 [−0.06, 0.06], −0.30 [−0.36, −0.25]; respectively) and females (*r* = −0.01 [−0.10, 0.09], 0.10 [0.01, 0.19], −0.12 [−0.21, −0.03], −0.01 [−0.08, 0.10]; respectively) (see Figure 3 and Figure 4). 

Females had a statistically significant correlation between BM and JH0 (*r* = −0.44 [−0.72, −0.04], *p* = 0.03) and males did not (*r* = −0.13 [−0.44, 0.21], *p* = 0.46). Neither males nor females produced a statistically significant correlation between JH20 and BM (*r* = 0.11 [−0.23, 0.43], −0.27 [−0.61,0.15]; *p* = 0.53, 0.20; respectively). However, IPFa in males produced statistically significant correlations between JH0 (*r* = 0.35 [0.02, 0.61], *p* = 0.04) and JH20 (*r* = 0.44 [0.13, 0.67], *p* < 0.01), whereas females produced no such relationship with IPFa (*r* = −0.03 [−0.43, 0.38], 0.00 [−0.40,0.40]; *p* = 0.89, 0.99; respectively). Additionally, males had a statistically significant correlation between PIAS20 and the percent difference between JH0 and JH20 (*r* = 0.35 [0.02, 0.61]; *p* = 0.04), whereas females did not reach statistical significance (*r* = 0.19 [−0.23, 0.55], *p* = 0.38). Interestingly, PIAS0 did not produce a statistically significant correlation between the percentage difference of JH0 and JH20 for males (*r* = 0.08 [−0.26, 0.40], *p* = 0.65) nor females (*r* = 0.16 [−0.26, 0.53], *p* = 0.35). 

## 4. Discussion

The purpose of this study was to investigate the relationship of asymmetry from both the propulsive phase and the positive impulse phase with jump height from unweighted and weighted jumps using collegiate athletes. In addition, the inclusion of both male and female athletes produced an auxiliary question of sex differences. These data illustrate the negative relationship of increasing asymmetry in the positive impulse phase has with CMJ performance. As well, a notable finding was that the propulsive phase impulse asymmetry does not produce statistically significant correlations with jump height performances. The negative relationship of PIAS and CMJ performance supported our hypothesis and illustrates that more symmetrical positive impulses produce greater total impulses leading to greater jumping performances. Although the literature is equivocal, the relationships between CMJ performance and PrPAS agree with previous findings investigating the propulsive phase [7]. Males and females have differing performance levels with similar asymmetry. This, combined with the ability of strength training to improve the coordination of muscle activation patterns [24,25], provides further evidence for the motor coordination aspect of asymmetry and the possibility of sport specific asymmetry development. 

Interpretation of these results does come with limitations as no injury data were made available for this study nor were currently injured soccer athletes assessed. Previous injuries may influence the relationship of asymmetry measures to performance even with all athletes being cleared by the athletic training staff. As such, athletes recovering from a recent injury, even though cleared to participate, may still display differing relationships to these variables [26]. Additionally, these data are applicable to collegiate soccer athletes and more research is needed to expand these findings to other levels of soccer athletes and differing sports.

Using PIAS is a novel approach to the asymmetry relationship to performance question and yields promising results that may clarify discrepancies previously published about asymmetry from dGRF. The propulsive phase alone is not a good indicator of CMJ performance asymmetry and should be combined with the braking phase. This stands to reason as the utilization of the stretch-shortening cycle influences the concentric contraction. Additionally, this may help explain the negligible relationship between CMJ asymmetry and isometric strength found in this study which is in line with previous investigations [5,27]. Differing motor demands elicit different asymmetry results. However, strength training has been shown to enhance muscle spindle utilization, increase reciprocal inhibition [28], and improve coordination [23,24]. Together, this suggests that dynamic strength measures that include an eccentric component could have a stronger relationship to CMJ asymmetry [3], especially during the positive impulse phase. It should also be noted that strength training specificity and degree of transfer to performance may play a role in altering asymmetry [25,29].

Asymmetry levels are similar between the sexes, but performances are not. The similar asymmetry between males and females may be explained by, and support the theory of, soccer specific asymmetry [30]. It is possible that previous studies investigating asymmetry using athletes from multiple sports experienced lower correlations due to the differing demands of each sport which may have differing asymmetry thresholds. With females having a high correlation between BM and jump heights and low correlations between IPFa and jumping performances, overall strength may have played a role in the lack of correlation between PIAS and jump performance in the weighted jumps. This indicates that the 20 kg selection for assessing weighted jump asymmetries may not be the most appropriate weight for individuals below a certain maximum strength level, though more research is needed in this area. These results are similar to disparities found between males and females in previous asymmetry research [13,31,32]. Overall, males seem to have better control of their body mass than females, which may be related to strength differences [5,31]. This is supported by males producing a greater correlation in PIAS20 to JH20 than PIAS0 to JH0.

Asymmetry is correlated between similar tasks and developing an asymmetry profile of an athlete from multiple tasks could theoretically be used to assist in understanding the overall implications asymmetry has for performance. Practitioners should use caution when comparing these results to other studies as the calculation used for assessing asymmetry does have an impact on assigning thresholds and recommendations. It is important to note that the current study implemented a calculation that yields lower asymmetry score results than studies that used a single limb or half of the sum of both limbs in the denominator. Assessing asymmetry is a simple addition to currently monitored dGRF profiles and may be used to spot unexpected adaptations in a longitudinal manner, though more research is needed in this area.

## 5. Conclusions

Coaches and practitioners should use caution when assessing asymmetry as the test and load needed may change depending on the population. Asymmetry alone accounts for only a portion of the variance in countermovement jump heights but does have a statistically significant negative correlation. It is recommended to assess asymmetry under both unloaded and loaded conditions when available. Additionally, asymmetry during the entire positive impulse phase should be used in the calculation of asymmetry instead of in the propulsive only phase.

## Figures and Tables

**Table 1:** Descriptive Data for Correlations.

	Males	Females
Body Mass (kg)	76.3 ± 7.8	68.8 ± 12.0
Height (cm)	178.3 ± 5.9	165.3 ± 19.5
Age	19.7 ± 1.5	19.5 ± 0.8
IPFa	189.6 ± 27.2	153.4 ± 25.4
JH0 (cm)	30.82 ± 4.25	21.44 ± 4.72
JH20 (cm)	22.19 ± 3.67	14.42 ± 3.19
PIAS0	7.10 ± 5.45	5.96 ± 5.55
PIAS20	7.06 ± 4.59	5.53 ± 4.25

Data presented as Mean ± SD; PIAS0 = Positive impulse asymmetry score for unweighted countermovement jumps; PIAS20 = Positive impulse asymmetry score for weighted (20 kg) countermovement jumps; IPFa = allometrically scaled isometric peak force; JH0 = unweighted jump height; JH20 = weighted (20 kg) jump height.

**Table 2:** Positive Impulse Asymmetry Score Correlations.

Description		Pearson Correlation (*r*) with [CI]	*R* ^2^	*p* Value
Females (*n* = 24)	Jump Height 0 kg: PIAS0	−0.43 [−0.71, −0.03]	0.19	0.03 *
	Jump Height 20 kg: PIAS20	−0.25 [−0.59, 0.17]	0.06	0.23
Males (*n* = 35)	Jump Height 0 kg: PIAS0	−0.32 [−0.59, 0.01]	0.10	0.06
	Jump Height 20 kg: PIAS20	−0.49 [−0.71, −0.19]	0.24	<0.01 *

CI = 95% confidence interval; JH0 = unweighted jump height; JH20 = weighted (20 kg) jump height; PIAS0 = Positive impulse asymmetry score for unweighted countermovement jumps; PIAS20 = Positive impulse asymmetry score for weighted (20 kg) countermovement jumps; * = Statistically significant with alpha of 0.05; All values based on LN transformation of PIAS0 and PIAS20.

**Table 3:** Propulsive Phase Asymmetry Score Correlations.

Description		Pearson Correlation (*r*) with [CI]	*R* ^2^	*p* Value
Females (*n* = 24)	Jump Height 0 kg: PrPAS0	−0.09 [−0.48, 0.33]	0.01	0.66
	Jump Height 20 kg: PrPAS20	0.11 [−0.31, 0.49]	0.01	0.60
Males (*n* = 35)	Jump Height 0 kg: PrPAS0	0.13 [−0.21, 0.44]	0.02	0.46
	Jump Height 20 kg: PrPAS20	0.26 [−0.08, 0.55]	0.07	0.13

CI = 95% confidence interval; JH0 = unweighted jump height; JH20 = weighted (20 kg) jump height; PrPAS0 = Positive impulse asymmetry score for unweighted countermovement jumps; PrPAS20 = Positive impulse asymmetry score for weighted (20 kg) countermovement jumps; Based on LN transformation of PrPAS0 and PrPAS20.

## Data Availability

Not applicable.
